# Isolated Spherophakia and Phacodonesis in a Young Child With Short Stature: A Case Report

**DOI:** 10.7759/cureus.81223

**Published:** 2025-03-26

**Authors:** Nicolas Nicolaou, Despina Nicolaou, Savvas Christou

**Affiliations:** 1 Ophthalmology, Faculty of Medicine and Dentistry, Queen Mary University of London, London, GBR; 2 Orthopedics, Addenbrooke's Hospital, Cambridge University Hospitals NHS Foundation Trust, Cambridge, GBR; 3 Ophthalmology, St George and Bluecross Hospital, Paphos, CYP

**Keywords:** acute angle closure, isolated spherophakia, lensectomy, lens subluxation, lenticular myopia, phacodonesis, progressive high myopia

## Abstract

Bilateral spherophakia is a rare congenital condition, typically associated with syndromic disorders where distinctive features facilitate early recognition. Isolated cases without systemic involvement are often underdiagnosed and identified following angle-closure glaucoma or crystalline lens (CL) subluxation. In spherophakia, the CL adopts a spherical shape due to defective zonular fibers, inducing lenticular myopia. We report the case of a six-year-old boy presenting with short stature, high myopia, and bilateral low vision, initially misdiagnosed. A progressive myopic shift of -0.50 D every three to six months led to a refractive error of -16.00 D over 10 years. Axial lengths, keratometry (K) readings, and posterior segment findings were normal, supporting a diagnosis of lenticular rather than axial or corneal myopia. Anterior chamber depths (ACD) and angles were bilaterally shallow. The co-existence of isolated spherophakia and short stature initiated genetic evaluation, given established associations with sporadic ADAMTS17 mutations. However, the results were inconclusive. Isolated spherophakia should be considered in children presenting with short stature and high myopia, particularly in consanguineous families. Grade 1 phacodonesis or lens hypermobility was observed on slit-lamp biomicroscopy, indicating CL instability. This report aims to increase awareness of spherophakia in the absence of systemic involvement. Key features include progressive high myopia with normal axial length, K readings, and increased CL thickness and power. Bilaterally shallow ACD and symptoms of intermittent blurred vision in dim light suggest angle closure. Bilateral amblyopia is also common. Careful observation for phacodonesis is emphasized as it is a potential risk for CL subluxation in spherophakia. Management strategies are outlined to support timely intervention.

## Introduction

Bilateral spherophakia is a congenital condition characterized by an abnormal spherical crystalline lens (CL) with increased anteroposterior thickness due to defective zonular fibers [1,2]. This structural abnormality leads to progressive lenticular myopia, while axial length, corneal curvature, and posterior segment remain unaffected [1,2]. Forward displacement of the lens increases the risk of pupillary block and angle-closure glaucoma (ACG) [3-5].

Spherophakia is frequently associated with systemic conditions linked to mutations in the LTBP2 and FBN1 genes, which encode essential zonular fiber proteins [6-8]. Occasionally, isolated cases emerge in early childhood but often remain undiagnosed, leading to bilateral amblyopia [3,8]. Short stature has been reported in some cases of isolated spherophakia without systemic involvement [9]. This has been observed in Weill-Marchesani-like syndrome, associated with recessive ADAMTS17 sporadic gene mutations [9].

We report a case of a six-year-old child with isolated bilateral spherophakia, short stature, and phacodonesis, initially misdiagnosed. The previously reported association between spherophakia and short stature prompted genetic and endocrine evaluation [9]. No definitive systemic cause was identified in this case, and the association remains a topic of debate. Nevertheless, the coexistence of short stature and isolated spherophakia should be considered in children presenting with progressive high myopia, particularly in consanguineous families where sporadic mutations have been documented [9]. Phacodonesis, or CL hypermobility, is a key clinical feature of spherophakia, reflecting zonular fiber instability [10,11]. In this case, Grade 1 phacodonesis was detected on slit-lamp examination.

The true prevalence of spherophakia remains unclear. In Cyprus, with a population of 1.2 million, only four cases have been documented, three syndromic and one isolated. However, some isolated cases may be underdiagnosed due to limited clinical recognition. This case raises awareness of lenticular myopia induced by spherophakia. Tracking its 10-year progression presents findings supporting timely identification of isolated cases and outlines key biometric markers. Additionally, it highlights the importance of continuous monitoring for phacodonesis, which may indicate CL subluxation, as well as assessment for ACG. Management of complications is also discussed.

## Case presentation

A six-year-old male, born at term with normal developmental milestones, presented with progressive high myopia. Cycloplegic refraction demonstrated a best vision sphere (BVS) of -5.00 diopters (D), with a best-corrected visual acuity (BCVA) of 6/12 in the right eye (OD) and 6/18 in the left eye (OS). The absence of strabismus, lens opacities, and normal posterior segment findings suggested that bilateral amblyopia was secondary to uncorrected myopia. An initial myopic shift of -1.50 D was documented over three months, followed by a sustained progression of -0.50 D every three to six months. The pattern was assumed to be due to axial myopia (Table 1).

**Table 1:** Summary of prescription changes from ages six to 16 years and corresponding diagnosis at different ages OD: oculus dexter (right eye), OS: oculus sinister (left eye), DS: diopters sphere, DC: diopters cylinder, D: diopters, BCVA: best-corrected visual acuity, ACD: anterior chamber depth, ACG: angle-closure glaucoma, CL: crystalline lens, K: keratometry

Age in years	Prescription of OD	Prescription of OS	Comments
	Sphere DS/cylinder DC × axis (D), BCVA (Snellen acuity)	Sphere DS/cylinder DC × axis (D), BCVA (Snellen acuity)	-
6	-4.50 DS/-0.75 DC x 109 (6/12)	-4.50 DS/-1.75 DC x 75 (6/18)	Myopia with bilateral amblyopia was found, with a normal posterior segment. No strabismus or lens opacities were present. Amblyopia developed due to uncorrected myopia.
6	-5.00 DS/-0.75 DC x 105 (6/12)	-6.00 DS/-2.00 DC x 74 (6/18)	Myopia increased by -0.5 0D every three to six months as the CL developed a spherical shape.
7	-5.50 DS/-0.50 DC x 110 (6/12)	-6.50 DS/-2.75 DC x 70 (6/18)	The myopic progression was initially attributed to axial elongation.
8	-6.75 DS/-0.50 DC x 90 (6/12)	-7.00 DS/-2.75 DC x 90 (6/18+2)	Normal axial length, K readings, and bilaterally shallow ACD supported a diagnosis of lenticular myopia secondary to spherophakia. Corneal astigmatism in the OS measured -5.75 D but was not corrected, as no visual improvement was noted. Corneal ectasia and keratoconus were not further evaluated, given the adequate corneal thickness observed on Oculus Pentacam (central corneal thickness: 744 µm; thinnest point: 718 µm).
9	-7.75 DS/-0.75 DC x 80 (6/12)	-7.50 DS/-2.75 DC x 80 (6/18)	Phacodonesis (Grade 1) was observed on slit-lamp examination, indicating lens hypermobility due to zonular instability. Grade 1 reflects a low to mild risk of lens subluxation.
10	-9.00 DS/-1.25 DC x 90 (6/12)	-9.00 DS/-3.00 DC x 80 (6/18)	Genetic testing for a systemic cause of spherophakia was undertaken due to the associated short stature. No definitive link was identified. A diagnosis of isolated bilateral spherophakia was confirmed.
10	-9.50 DS/-1.50 DC x 100 (6/12)	-9.50 DS/-3.00 DC x 80 (6/18)	-
13	-14.50 DS/-1.50 DC x 105 (6/12)	-14.50 DS/-3.00 DC x 80 (6/18)	Continuous monitoring for ACG and CL subluxation. No intermittent episodes of pupillary block were observed.
16	-15.50 DS/-1.50 DC x 100 (6/12)	-15.50 DS/-3.00 DC x 100 (6/24+3)	Discussions held for optimal prescription correction. Refractive laser correction is not advised.

**Figure 1:**
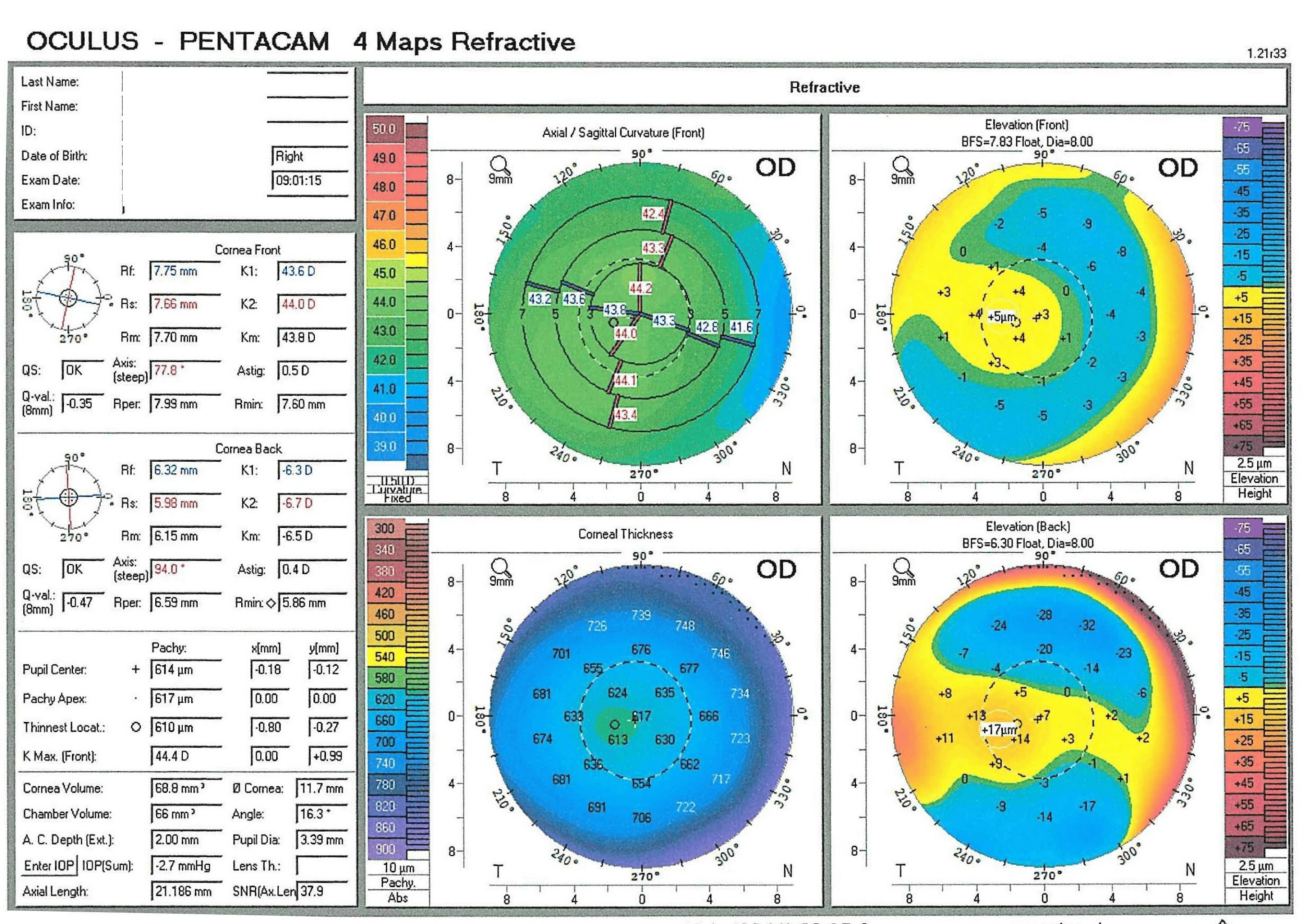
Oculus Pentacam refractive maps of OD at age eight years Pentacam measurements in spherophakia reveal a normal axial length (21.186 mm OD) and normal K readings (K1, K2), a shallow ACD of 2.00 mm, and a reduced anterior chamber angle of 16.3°. These findings are consistent with lenticular myopia. Note: Lens thickness, typically increased in spherophakia, is not measured by the Oculus Pentacam. Elevation maps relative to best-fit spheres (anterior: 7.83 mm; posterior: 6.30 mm) demonstrate no signs of ectasia, with maximum elevations of +5 µm (anterior) and +17 µm (posterior), confirming a normal corneal structure. A central corneal thickness of 610 µm is atypical for keratoconus and makes the diagnosis less likely. Normal reference ranges: K values (IQR: 43.6-44.78 D), axial length (IQR: 22.15-23.00 mm), ACD ( 3.0-3.50 mm), and anterior chamber angle (35.9 ± 5.7°) [12] OD: oculus dexter (right eye), K: keratometry, D: diopters, ACD: anterior chamber depth, IQR: interquartile range Zhu et al., 2024 [12]

**Figure 2:**
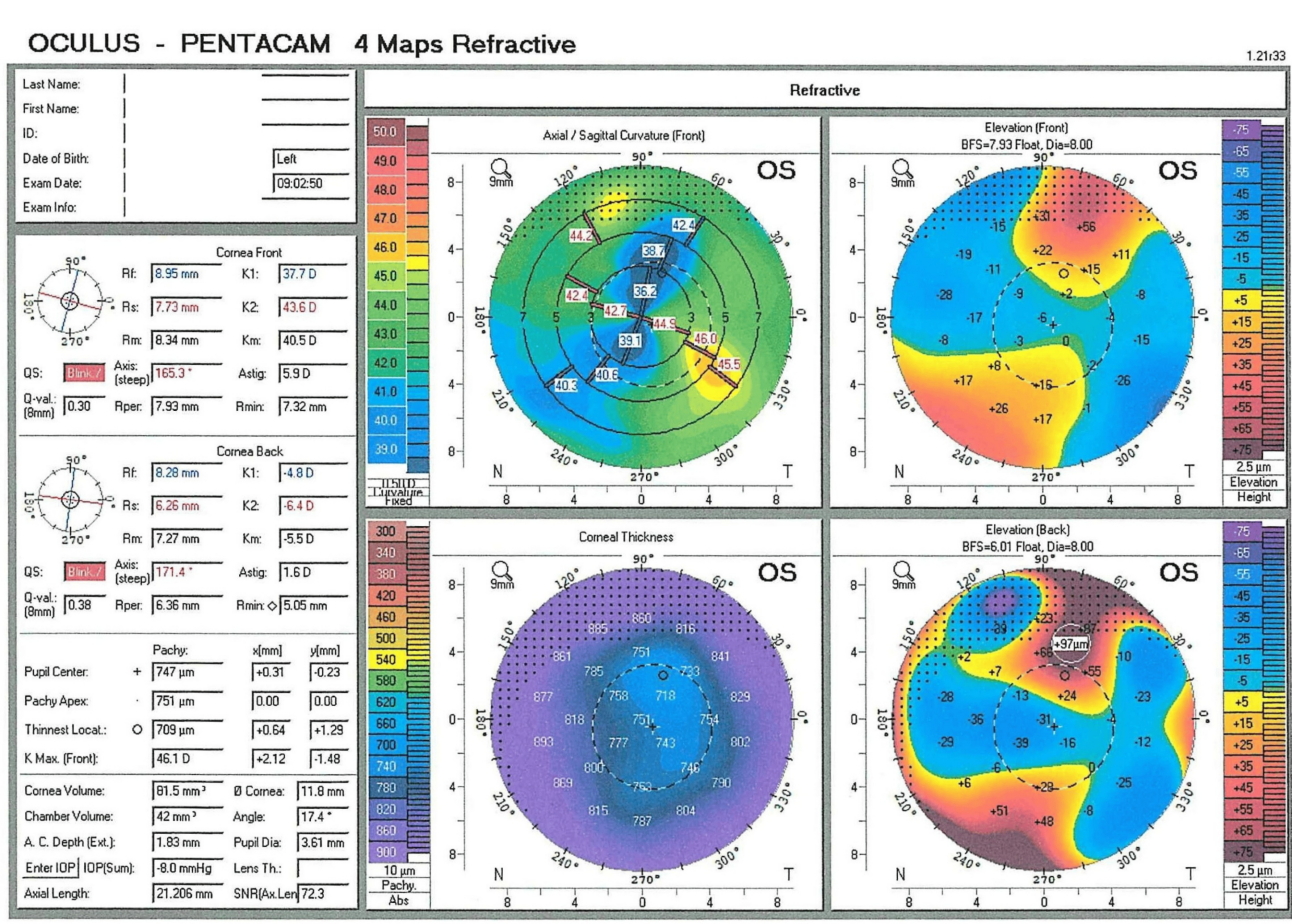
Oculus Pentacam refractive maps of OS at age eight years Axial length was normal, indicating lenticular myopia. K values (K1: 37.7 D, K2: 43.6 D) indicated high astigmatism (-5.75 D), with a slightly steep Kmax of 46.1 D. The axial curvature map revealed an irregular pattern, while both the anterior and posterior elevation maps showed abnormal elevations (+56 µm and +97 µm, respectively). However, corneal pachymetry demonstrated a thick, symmetrically distributed cornea (central: 744 µm; thinnest: 718 µm), which was inconsistent with corneal ectasia. ACD was shallow (1.83 mm), and the anterior chamber angle was narrowed to 17.4°, suggesting an increased risk of angle closure. Normal reference ranges: K values (IQR: 43.6–44.78 D), axial length (IQR: 22.15–23.00 mm), ACD (3.0–3.50 mm), and anterior chamber angle (35.9° ± 5.7°) [12] OS: oculus sinister (left eye), K: keratometry. D: diopters, ACD: anterior chamber depth, IQR: interquartile range Zhu et al., 2024 [12]

Further investigations using Optovue spectral-domain optical coherence tomography (Optovue, Inc., North Lombard, IL, USA) with radial line scans provided high-resolution cross-sectional images of the macula and optic nerve head, revealing normal posterior segments. Oculus Pentacam (OCULUS, Wetzlar, Germany) revealed normal axial lengths of 21.186 mm OD and 21.206 mm OS, indicating refractive instead of axial myopia (Figures 1-2). However, keratometry (K) values of 43.6/44.0 D OD and 37.7/43.6 D OS were normal, suggesting lenticular rather than corneal-induced myopia, which would typically present with higher values [12]. OS was noted to have -5.75 D astigmatism, raising concerns for corneal ectasia. However, the pachymetry map showed a thick cornea (744 µm at the pupil center and 718 µm at its thinnest point), inconsistent with corneal ectasia (Figure 2). Given this finding, a Belin/Ambrosio Enhanced Ectasia Display analysis was not further evaluated then. The increased corneal thickness was attributed to a congenital variation, as no signs of corneal dystrophy or edema were observed (Figures 1-2).

**Figure 3:**
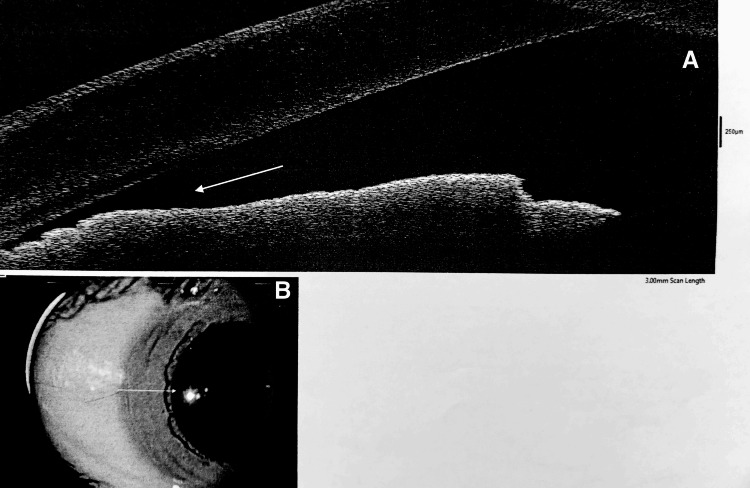
AS-OCT A: High-resolution AS-OCT scan showing a narrow anterior chamber angle (white arrow) in OD. B: Infrared image of OD corresponding to the AS-OCT scan. The arrow indicates the location of the anterior chamber angle, where narrowing is observed. AS-OCT: anterior segment optical coherence tomography, OD: oculus dexter (right eye)

**Figure 4:**
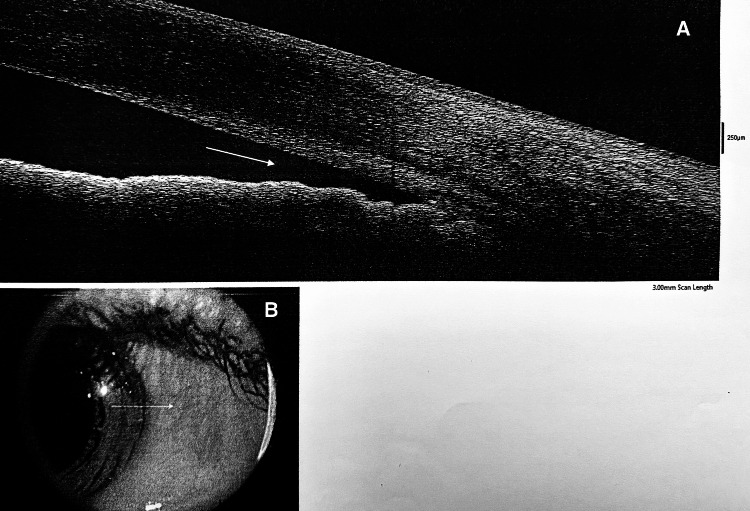
AS-OCT A: High-resolution AS-OCT scan of the anterior segment of OS, showing a narrow anterior chamber angle (white arrow). Bilateral findings, such as shallow ACD and narrow angles associated with high myopia, suggest anterior displacement of a spherical CL with increased anteroposterior thickness. B: Infrared image of OD corresponding to the AS-OCT scan. The white arrow indicates the location of the anterior chamber angle where narrowing is observed. AS-OCT: anterior segment optical coherence tomography, ACD: anterior chamber depth, CL: crystalline lens, OS: oculus sinister (left eye), OD: oculus dexter (right eye)

The Pentacam revealed bilaterally shallow ACDs and narrow angles (Figures 1-2), while anterior segment optical coherence tomography (AS-OCT) provided clearer visualization of the narrow angles (Figures 3-4). Many reported cases were identified after an elevation in intraocular pressure (IOP) due to angle closure, which facilitated the recognition of spherophakia [4,7]. In this case, however, IOP was within normal limits (18 mmHg OD, 17 mmHg OS), which contributed to delayed diagnosis (increased corneal thickness can lead to overestimation of IOP measurements).

Based on shallow ACDs, normal axial lengths, K, and rapid myopic progression, a pediatric ophthalmologist diagnosed lenticular myopia secondary to spherophakia. While the Oculus Pentacam does not record lens thickness, the spherical appearance on slit-lamp examination further supported the diagnosis. A-scan biometry can estimate CL thickness, while ultrasound biomicroscopy offers more precise measurements.

By age nine, significant progression in refractive error was recorded, with a BVS of OD -8.00 diopters sphere (DS) (BCVA: 6/12) and OS -9.00 DS (BCVA: 6/18) (Table 1). Further slit-lamp observations revealed a mild tremor of the CL during eye movements, consistent with (+1) phacodonesis. This finding increases the risk of CL subluxation and necessitates regular monitoring. The presence of short stature and spherophakia also prompted further pediatric and genetic evaluations to assess potential syndromic involvement. Endocrine assessment of growth hormone levels for short stature was within normal limits.

Genetic testing via whole-exome sequencing identified a COL11A1 (collagen type XI alpha 1 chain) gene variant (c.4032G>A, p.Pro1344=; NM_001854.4), associated with Stickler syndrome type 2 (STL2), an autosomal dominant heterozygous disorder. This variant was classified as having unknown significance. Importantly, the patient exhibited no hallmark features of STL2, such as congenital vitreous anomalies, congenital megalophthalmos, sensorineural or conductive hearing loss, brachydactyly, high-arched palate, or arthropathy [13]. Genetic testing of family members identified the same variant in the father, who was asymptomatic and of normal height, indicating that the variant was not a de novo mutation. These findings led to a revised diagnosis of isolated bilateral spherophakia. Additionally, the corneal diameter measured 11.8 mm, effectively ruling out megalocornea associated with syndromic conditions.

IOP consistently remained below 18-20 mmHg. No episodes of blurred vision in dim lighting were reported. Prophylactic measures, including the potential use of peripheral iridotomy and cholinergic eye drops, were considered in case of future IOP elevation. At age 14, the patient’s height was 1.53 m. With genetic evaluation ruling out syndromic associations, the short stature was attributed to family traits, further confirming isolated spherophakia. Refractive error had progressed to a BVS of -14.50 D in both eyes, with a BCVA of OD 6/12 and OS 6/18 (Table 1). Further visual acuity improvement was not achievable with glasses or specialized contact lenses. The patient reported intermittent diplopia and difficulty sustaining near vision for extended periods.

The current management strategy focuses on regularly updating prescriptions to optimize vision and close monitoring for potential complications. Discussions regarding myopia correction presented challenges. Refractive laser surgery was contraindicated due to ongoing spherical changes in the CL, leading to unpredictable myopia progression. The unstable nature of lenticular myopia, which typically ranges from -10.00 D to -15.00 D as observed in this case, is evident in extreme cases where myopia has progressed up to -28.00 D [1,4]. Surgical intervention was also not indicated at this stage, as no ACG or lens subluxation episodes were presented.

## Discussion

Spherophakia is a rare congenital bilateral condition, most often associated with syndromic disorders in pediatric cases. Isolated presentations within the first decade of life are even rarer and frequently underrecognized [1,2]. It results from weakened or hypoplastic zonular fibers, which normally support the lens. Consequently, the lens adopts a progressively spherical shape with increased anteroposterior thickness [1]. The highly convex lens refracts the focal point anterior to the fovea, leading to lenticular myopia [1,3]. Unlike axial myopia, which progresses more gradually, lenticular myopia is marked by more frequent changes.

Biometric and clinical features can differentiate lenticular myopia from axial myopia shown in Table 2. High myopia with a progressive myopic shift every three to six months, along with normal axial length and corneal curvature, is a hallmark feature [1,2]. The posterior segment is typically normal. Most cases exhibit bilaterally reduced ACDs of less than 2.50 mm as reported by Khokhar et al. and Zhu et al. [1,12]. While shallow ACD and ACG are more commonly associated with hyperopia, their presence in highly myopic eyes should prompt evaluation for spherophakia. Increased lens thickness and anterior displacement of the CL contribute to angle narrowing and elevated IOP [4,5].

**Table 2:** Key biometric and clinical features of bilateral spherophakia Hallmark findings aiding the recognition of spherophakia include progressive myopia with normal axial length and K values, along with increased CL thickness and power within the specified ranges. ACD is typically shallow (<2.5 mm) and bilateral in most cases due to forward displacement of the spherical CL [1-3,12]. Additional features may include bilateral amblyopia, intermittent pupillary block, raised IOP, and ACG [1-3]. * Phacodonesis, detectable on slit-lamp examination, indicates zonular instability and an increased risk of lens subluxation. The phacodonesis grading system is as follows: Grade 1: Mild phacodonesis, detectable on careful slit-lamp examination, with no iridodonesis (iris tremor) or zonular dialysis (zonular detachment). Grade 2: Moderate phacodonesis, easily visible, with or without iridodonesis, but without zonular dialysis. Grade 3: Severe phacodonesis with iridodonesis, zonular dialysis, and possible lens dislocation. Grade 3 indicates a higher risk of CL subluxation [10]. CL: crystalline lens, ACG: angle-closure glaucoma, BCVA: best-corrected visual acuity, IOP: intraocular pressure, IQR: interquartile range, K: keratometry Khokhar et al., 2018 [1]; Mohamed et al., 2017 [2]; Kannan et al., 2020 [3]; Liu et al., 2011 [10]; and Zhu et al., 2024 [12]

Feature type	Description
Hallmark features	
High progressive myopia	-
Normal axial length	IQR: 21.23–23.59 mm [1,12]
Normal keratometric values	IQR: 42.88–45.45 D [12]
Normal fundus	-
Shallow ACD	<2.50 mm [1,2,12]
Increased CL thickness	>4.26 mm [2]
Increased CL power	IQR 34.54-46.51 D [12]
Additional features	
Elevated IOP	-
ACG	-
Intermittent pupillary block	Blurred vision in dim light or prone position
Phacodonesis (hypermobility of CL)	*
Bilateral amblyopia	Reduced BCVA [3]
CL subluxation	-

Lens thickness and power are key diagnostic parameters in spherophakia (Table 2). Studies report that lens thickness frequently exceeds the normal range of 3.89-4.26 mm, reaching up to 5.84 mm [2]. Lens power serves as a reliable indicator. In a study of 165 spherophakic eyes, the IQR was 34.54-46.51 D, with a sensitivity of 94.3% and specificity of 91.9% [12]. When direct measurement is unavailable, the CL power can be estimated using the online SRK-T formula, which incorporates K, axial length, ACD measurements, and an A-constant of 118.4 [12]. In this case, the calculated CL power was +29.00 D in OD and +32.00 D in OS. Although these values fall below the proposed spherophakia range, they remain higher than those observed in high axial myopia (IQR: 18.00-25.50 D) and normal eyes [12].

Bilateral amblyopia is a common presentation in spherophakia, as observed in our case and other reports, due to delayed diagnosis of lenticular myopia in early childhood [3-5,12]. This finding aligns with Khokhar et al., where all 26 spherophakic eyes exhibited BCVA ranging from 6/12 to 1/60 [1]. Similarly, Kannan et al. found that among 32 eyes undergoing surgery, with a median age of 19.5 years, BCVA ranged between 6/18 and 6/60 [3]. A highly myopic child with persistent bilateral low vision beyond the critical window, particularly with progressive refractive changes and normal axial length, should be evaluated for spherophakia [3]. Table 2 highlights key diagnostic features and optical parameters essential for diagnosing isolated spherophakia.

The CL's increased curvature and forward bulging, particularly at its periphery, contribute to trabecular meshwork crowding, predisposing to peripheral anterior synechiae, pupillary block, and ACG. Intermittent pupillary block can cause episodic IOP elevation, leading to blurred vision in dim light or a prone position (Table 2). This should raise suspicion of narrow angles and prompt further investigation [3-5].

In this patient, cholinergic eye drops were considered a potential intervention alongside Nd:YAG laser peripheral iridotomy in the event of elevated IOP or intermittent pupillary block. However, mitotic agents may increase iris-lens contact and exacerbate pupillary block. In contrast, cycloplegic agents, which relax the ciliary muscle and tighten the zonular fibers, can move the lens posteriorly, thereby widening the anterior chamber angle [4]. Additionally, topical β-blockers reduce aqueous humor production [5].

Phacodonesis of the CL may occasionally be observed on slit-lamp biomicroscopy. In this case, Grade 1 phacodonesis was noted during eye movement. Clinicians should assess for hypermobility in cases of high myopia with normal axial length, as it may indicate zonular fiber instability and potential CL subluxation. Phacodonesis is graded on a scale of 1 to 3, with higher grades indicating greater risk, as explained in Table 2 [10,11].

Subluxation of the CL has been reported in both isolated and systemic cases [1,4,5]. Mohamed et al. found that the CL in spherophakia has an increased wet weight, approximately 53% greater than that of a normal lens [2]. The increased lens weight and zonular fiber weakness contribute to lens dislocation or subluxation, which may occur anteriorly or posteriorly, leading to angle closure [1].

Bilateral spherophakia can be subdivided into two categories. The most common type is associated with syndromic conditions. Zonular fibers primarily comprise FBN1 (64%) and LTBP2 proteins [6]. Mutations in FBN1 are linked to autosomal dominant Weill-Marchesani syndrome and Marfan syndrome. Autosomal recessive mutations in ADAMTS10, ADAMTS17, and LTBP2 have been implicated in these syndromes [7-9]. Other associated conditions include homocystinuria, Alport syndrome, Ehlers-Danlos syndrome, Klinefelter syndrome, and Axenfeld-Rieger syndrome [1,3].

The second category includes isolated spherophakia, though the cause remains unclear. While systemic features are absent in isolated cases, an underlying genetic origin may still exist. Khan et al. identified three distinct recessive ADAMTS17 mutations in children with Weill-Marchesani-like syndrome, who presented with isolated spherophakia and short stature [9]. While these children had short stature, they did not exhibit brachydactyly, a high-arched palate, joint stiffness, or other congenital abnormalities. The authors concluded that three variants of this gene, which we should be aware of, are associated with isolated spherophakia with proportionate short stature. High myopia and short stature should be evaluated for spherophakia, particularly in consanguineous families. Sporadic mutations have also been linked to LTBP2 variants, which cause zonular fiber weakness without systemic manifestations in isolated cases [8].

While genetic testing does not directly contribute to managing isolated spherophakia, it provides valuable insights into underlying causes and emerging associations. Detecting such genetic links enhances clinical awareness and improves early recognition of related conditions. For instance, Sturge-Weber syndrome has recently been reported in association with isolated spherophakia, a previously unknown connection [14]. Identifying such associations through genetic investigation may refine the diagnostic approach and heighten suspicion in new cases.

A variant of STL2 was detected in our case. Spherophakia was not previously reported in STL2, a connective tissue disorder caused by COL11A1 mutations. STL2 is more commonly associated with high axial myopia rather than lenticular myopia [13]. While this variant was classified as of unknown significance, its potential role as a disease-causing mutation could not be excluded, as recorded in the ClinVar database. Given its involvement in connective tissue disorders, further research is needed to explore a possible association between STL2 and spherophakia.

**Figure 5:**
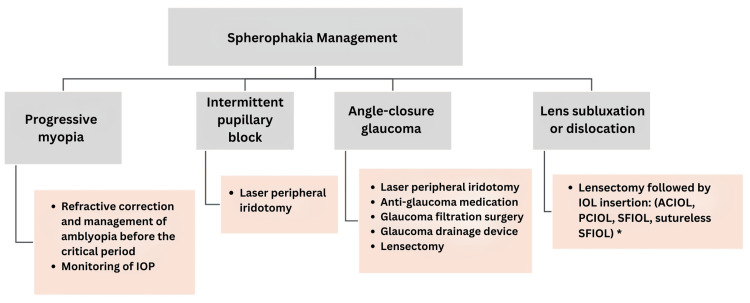
Management of spherophakia with and without complications * Types of IOLs used for lens subluxation and dislocation: benefits and risks [3,15] ACIOLs effectively manage aphakia, providing good visual outcomes with minimal endothelial damage. Risks: Chronic uveitis, peripheral anterior synechiae, glaucoma, iris chafing, pupillary constriction, and long-term instability [3]. PCIOLs offer good centration if zonular support is adequate. Risks: A high risk of bag-lens complex dislocation into the vitreous due to weak zonules [3]. SFIOLs ensure stable IOL placement without the capsular bag, minimizing anterior segment crowding. There are two primary methods: Sutured SFIOLs provide long-term stability but may lead to complications such as suture breakage, IOL decentration, retinal detachment (4.9%), and cystoid macular edema (7.3%). Sutureless SFIOLs avoid suture-related complications by using scleral tunnels or glue, but require precise technique to prevent IOL tilt or decentration [3]. Carlevale SFIOL and CM T Flex SFIOL are excellent sutureless options for aphakia, offering high stability and a low risk of decentration. The Carlevale SFIOL utilizes flexible sclerocorneal plugs, whereas the CM T Flex SFIOL features T-shaped haptics anchored in scleral tunnels. Carlevale carries a small risk of haptic exposure, while CM T Flex minimizes this risk through its tunnel-based anchoring design. Due to its anchoring method, CM T Flex may also have a lower risk of plug externalization and pseudophakodonesis. While both require careful surgical technique, the CM T Flex SFIOL offers a simplified implantation process and potentially reduces complications [15]. IOLs: intraocular lenses, ACIOLs: iris-supported anterior chamber intraocular lenses, PCIOLs: posterior chamber intraocular lenses, SFIOLs: scleral-fixated intraocular lenses Kannan et al., 2020 [3] and Madhivanan et al., 2024 [15]

The management of spherophakia, as outlined in Figure 5, involves continuous monitoring and correction of progressive myopia in uncomplicated cases and laser peripheral iridotomy in the presence of intermittent pupillary block. Surgical intervention is required in cases of ACG or subluxation of the CL. The success of surgical management and the risk of postoperative lens dislocation are influenced by underlying systemic conditions, particularly connective tissue disorders such as Marfan syndrome [1-3]. Due to variability in zonular laxity, a tailored surgical approach is necessary, as no single procedure is universally applicable. In-the-bag IOL implantation may be effective in uncomplicated cases, but patients with collagen disorders face a higher risk of future dislocation due to inherent zonular weakness [1,3].

Lensectomy is commonly performed for lens subluxation or ACG; however, this procedure often results in high uncorrected refractive error, requiring customized contact lenses [1,2]. Scleral-fixated IOLs (SFIOLs) are currently the preferred correction methods [3,15]. A newly designed sutureless SFIOL, the CM T Flex, similar to the Carlevale SFIOL, was recently evaluated in a study involving 57 eyes. The findings demonstrated fewer cases of IOL haptic exposure, IOL dislocation, and complications such as cystoid macular edema, suggesting improved outcomes for patients requiring SFIOL implantation. However, the study was small [15]. As these interventions are relatively new, further prospective long-term studies on potential complications are still needed. Ongoing research will help refine clinical decision-making and optimize patient outcomes in SFIOL implantation.

## Conclusions

Lenticular myopia due to spherophakia remains underrecognized, especially when it presents in isolation. In syndromic cases, distinctive features often facilitate early diagnosis, whereas isolated bilateral spherophakia without complications is more challenging. Clinicians should consider isolated spherophakia in patients with high myopia and short stature, particularly in the context of consanguinity. Genetic evaluation can aid in identifying new associations or sporadic mutations, improving early detection of isolated cases. While STL2 has not yet been associated with spherophakia, ongoing case identification may provide new insights. This case highlights additional features that may facilitate the diagnosis of isolated spherophakia, with bilateral signs particularly important in raising clinical suspicion. Frequent myopic shifts in the presence of normal axial lengths and K values are key findings. Shallow ACDs and increased lens thickness and power in both eyes are characteristic signs. Bilateral amblyopia is common with high myopia. Phacodonesis, often missed, should be assessed carefully on slit-lamp examination, as early detection increases vigilance for potential lens subluxation. Intermittent blurred vision in dim light and elevated IOP require close monitoring for ACG. Early recognition and regular follow-up are essential for timely intervention.
